# Toxic Epidermal Necrolysis: A Case Report on a Drug-Induced Phenomenon

**DOI:** 10.7759/cureus.30407

**Published:** 2022-10-17

**Authors:** Soumya Pamnani, Sanket S Bakshi, Sourya Acharya

**Affiliations:** 1 Medicine, Jawaharlal Nehru Medical College, Datta Meghe Institute of Medical Sciences (Deemed to be University), Wardha, IND

**Keywords:** stevens-johnson syndrome (sjs), drug reaction, inflammation, carbamazepine, toxic epidermal necrolysis

## Abstract

Toxic epidermal necrolysis (TEN) is a group of severe forms of several life-threatening conditions. As a co-infection of this group, Stevens-Johnson syndrome (SJS) is a rare though severe disease of the skin and mucous membranes. Intake of some drugs could cause reactions such as SJS and TEN. A form of severe connective tissue disorder, TEN is also known as Lyell's syndrome and is a common cause of significant skin and mucous membrane disintegration. Adverse medication reactions are the most prevalent and contribute highly to the incidence rates of the major etiological variables for TEN. Erythema, epidermal detachment that manifests as blisters, and denuded skin patches are the defining features of this pathology. In the majority of cases, the administration of pharmaceutical drugs is thought to be the primary cause of SJS/TEN. In this article, we report a case of a 33-year-old male patient who presented with complaints of lower left facial pain and thus was prescribed carbamazepine. Following this, the patient presented with an adverse reaction to the administration of carbamazepine and was taken off the drug immediately. The treatment included the administration of hydration therapy and appropriate antibiotics for treating the fluid-filled vesicles. The treatment regimen continued for three weeks and was stopped when the skin lesions were scarce and there was an improvement in the overall health of the patient.

## Introduction

Stevens-Johnson syndrome (SJS) and toxic epidermal necrolysis (TEN) are generally regarded as distinct presentations of the same disease by authors and experts [1]. The Scottish dermatologist Alan Lyell first used the term TEN to describe a rare critical disorder known as Lyell's syndrome in 1956 [2]. Toxic epidermal necrolysis is frequently associated with widespread blisters emerging as macules and/or flat atypical targets wherever there is a significant detachment of mucous membrane and skin shown by full-thickness necrosis of the epidermis [3]. Additionally, they are severe, episodic, acute mucocutaneous reactions that are most often brought on by medications and seldom by infections [4]. The overlap between SJS and TEN is defined as dermal detachment between 10% and 29%. Toxic epidermal necrolysis involves more than 30% epidermal detachment, whereas SJS involves less than 10%. Anticonvulsants, non-steroidal anti-inflammatory drugs, sulfonamides, and antibiotics are a few of the drugs that might result in TEN [5]. Anticonvulsants are one of the primary causes of SJS/TEN, and among them, carbamazepine is responsible for the majority of the instances [6,7]. Originally used to treat epilepsy, the anti-epileptic, iminostilbene derivative carbamazepine is now used in increasing frequency for a variety of indications, including chronic pain, tic douloureux, and herpetic neuralgia [8]. Based on the observation that TEN always manifests one to three weeks after the injection of the suspicious drug, the relationship between TEN and drug use can be understood. Until the epithelium regenerates, isolation, hydration and electrolyte balance, nutritional support, pain control, and protective dressings are the most important auxiliary measures [9].

## Case presentation

**Figure 1:**
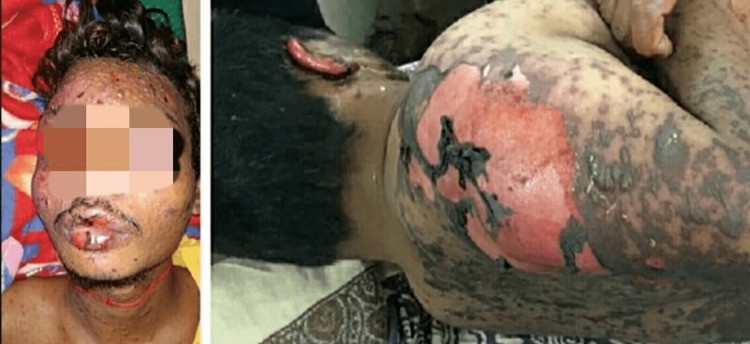
The dermatological manifestations of the reaction to the suspected drug, carbamazepine.

The main complaint of a 33-year-old male patient who visited the dermatology department was pain in the lower left facial area that had been present from the previous month. The lower facial region of one side was severely tender and the pain was piercing in nature. The pain was brought on by chewing, biting, or touching the affected areas of the face. The following week, the administration of carbamazepine was initiated and oral prophylaxis was also prescribed at the same time. At his subsequent appointment, the patient once more complained of pain in the same location. We chose to continue the same course of treatment while increasing the dosage of carbamazepine. The patient developed a quickly progressing, generalized eruption on day 14 after starting the prescribed drugs, along with a mild temperature, malaise, sore throat, cough, diarrhea, and pain. Following an inspection of the cheek, face, forearm, and lower extremities, severe bullae and oral rashes were discovered. The patient was then admitted, and the following medications were administered as part of his initial treatment: intravenous fluids (normal saline and Ringer's lactate), intramuscular injection pheniramine, and intravenous dexamethasone 8 mg thrice a day were prescribed. The following day, the patient was moved to an isolation area. The random blood sugar level was 197 mg/dl. Blood counts and urinalyses were within normal limits, while chest X-ray and electrocardiogram (ECG) results showed no alterations. On the lips, there were severe erosions and bullae, as well as broken bullae on the cheek, neck, forearm, and leg. In addition to having a sore throat, the patient had trouble swallowing. After the rashes progressed to the higher limbs, trunk, and legs, the patient developed fluid-filled sores all over his body, including around the eyes and lips (Figure 1). An epidermal detachment covering more than 70% of the body surface area and diffuse global skin peeling were both found during the clinical examination of his skin (also seen in Figure 1). It was easy to see Nikolsky's sign, which is epidermal separation brought on by slight lateral pressure on the skin's surface. As a result of the mucous membrane congestion, mucopurulent discharge, and exposure keratitis, toxic epidermal necrolysis with spots was diagnosed using the Bastuji classification [10].

The suspected medicine, carbamazepine, was withdrawn. An application of gentian violet paint was used to treat mouth ulcers. To prevent the skin from clinging to the cotton bed, the patient was forced to adjust his position on a sterile sheet. Topical antibiotic medicines such as topical gentamicin, moxifloxacin eye drops, and ocular lubricant solution were used to treat the ocular lesions. Supportive care included protein supplements for maintaining nutrition, and a stringent diet was prescribed by a nutritionist for the first 10 days until he switched to conventional regimens after the lesions began to heal. Dexamethasone was gradually reduced and then discontinued after seven days. By the third week of the sickness, the lesions had healed. The progression of the skin lesions stopped during the third week, and the patient's overall health improved.

## Discussion

Trigeminal neuralgia causes excruciating, lancinating pain that can be brought on by any stimulus [2,11]. Anticonvulsants are the drugs that are used to treat painful trigeminal neuralgia symptoms, and carbamazepine, one of the most frequently prescribed drugs associated with hypersensitivity reactions and closely associated with TEN, is the first-line treatment. Having said that, anticonvulsants like diphenylhydantoin, barbiturate, and carbamazepine have an 11% to 15% relative chance of causing SJS/TEN [12]. The causes of SJS and TEN are several. However, the most common cause appears to be medication [13,14]. Both SJS and TEN are now thought to have the same disease mechanism, with the skin surface area being the sole difference [15]. A minor version of TEN is one in which less than 10% of the body surface area (BSA) is involved; 10% to 30% of the BSA overlaps with SJS/TEN, and involvement of more than 30% of the BSA is regarded as TEN [16].

According to the criteria by Roujeau et al., alternative factors, such as infections, had to be cleared out before a drug could be blamed for an adverse drug reaction that occurred less than three weeks after the medicine was started [17]. After starting the treatment with carbamazepine, our patient had extensive skin peeling along with mucosal ulcers. Less than three weeks passed between the time carbamazepine was started and the onset of TEN, which is consistent with earlier findings [18]. Cutaneous symptoms started two weeks after the carbamazepine was started. Clinical symptoms of the patient were fever, malaise, sore throat, burning of the mucous membranes, and myalgias. About 24 hours later, skin lesions with diffuse macular erythema and targeted lesions started to develop. These lesions can result in huge, flaccid bullae that grow into larger ones [19]. Mucosal surfaces, including the lips, mouth, and conjunctiva, were severely affected. This was determined to be TEN, which is almost usually caused by drugs due to the persistent clinical signs and the fact that more than 70% of the body area was affected. There is no widely established, unquestionably successful, or targeted treatment for SJS/TEN other than confirmative care; the major therapeutic activity in TEN is early detection of the drug reaction and withdrawal of that particular substance [20]. Confirmative care is an essential component of its therapeutic strategy because it may be a life-threatening condition. Acid-base and metabolic balance regulation, fluid and electrolyte replacement, glucose management, and topical skin treatment comprise the initial administration. Frequent mouthwashes are advised to treat oral lesions.

The patient can drink fluids and experience less discomfort as a result of the administration of topical anesthetics. Covering any exposed skin with sterile cloths that have been embedded with paraffin or saline is advised. The use of general corticosteroids is controversial since they may prevent an immune reaction to the medication but may also encourage infection after the epidermis sheds. An initial dose of 30 to 40 mg per day of dexamethasone was given for several days before being tapered off, and this helped to reduce the healing time by 10 days, especially when treatment is initiated early in the course of the disease, as was the scenario in our case. The patient's full recovery was probably attributed to the prompt and successful management of inflammation with systemic steroid therapy and the appropriate topical care of the scouring regions. According to past research, systemic steroids, antibiotics, and adjunct therapies were used to treat the majority of TEN patients [21]. Careful handling, nutritional support, aggressive fluid and electrolyte control, and pain management are all required. The cornerstone of the treatment may be the importance of sterile wound care. A sterile dressing should be applied often to necrotic tissue. As a wound dressing, a variety of biological and artificial compounds are used with varying degrees of success. To prevent infections, we frequently clothe our patients with inseminated paraffin gauze. It is important to note that individuals with a history of SJS or a unique reaction to carbamazepine should not receive the medicine again, and a complete medical history should be collected before any prescriptions are written. Our patient's condition improved after carbamazepine was stopped, therefore drug withdrawal is the first line of treatment for medication-induced TEN. The early and successful treatment of our patient with general antibiotics and steroids, as well as the acceptable topical care of the scouring regions with inseminated paraffin sterile dressings, were most likely responsible. 

## Conclusions

Toxic epidermal necrolysis must be treated in burn centers as they have the necessary expertise to effectively handle the complications associated with severe skin loss. The pathophysiology of the illness is similar to that in patients with severe burns (fluid loss, risk of multiple organ dysfunction, risk of sepsis). Specific pharmacological treatment is not strongly advised due to a lack of strong evidence.
